# Honokiol as a specific collagen receptor glycoprotein VI antagonist on human platelets: Functional ex vivo and in vivo studies

**DOI:** 10.1038/srep40002

**Published:** 2017-01-05

**Authors:** Tzu-Yin Lee, Chao-Chien Chang, Wan-Jung Lu, Ting-Lin Yen, Kuan-Hung Lin, Pitchairaj Geraldine, Jiun-Yi Li, Joen-Rong Sheu

**Affiliations:** 1 Graduate Institute of Medical Sciences and Department of Pharmacology, College of Medicine, Taipei Medical University, Taipei, Taiwan; 2 Division of Cardiology, Department of Internal Medicine, Cathay General Hospital, Taipei, Taiwan; 3 Department of Medical Research, Taipei Medical University Hospital, Taipei, Taiwan; 4 Central Laboratory, Shin-Kong Wu Ho-Su Memorial Hospital, Taipei, Taiwan; 5 Department of Animal Science, School of Life Sciences, Bharathidasan University, Tiruchirappalli, Tamil Nadu, India; 6 Department of Cardiovascular Surgery, Mackay Memorial Hospital, and Mackay Medical College, Taipei, Taiwan

## Abstract

Honokiol, derived from *Magnolia officinalis*, has various pharmacological properties. Platelet activation plays a critical role in cardiovascular diseases. Honokiol has been reported to inhibit collagen-stimulated rabbit platelet aggregation. However, detailed further studies on the characteristics and functional activity of honokiol in platelet activation are relatively lacking. In the present study, honokiol specifically inhibited platelet aggregation and Ca^+2^ ion mobilization stimulated with collagen or convulxin, an agonist of glycoprotein (GP) VI, but not with aggretin, an agonist of integrin α_2_β_1_. Honokiol also attenuated the phosphorylation of Lyn, PLCγ2, PKC, MAPKs, and Akt after convulxin stimulation. Honokiol have no cytotoxicity in zebrafish embryos. Honokiol diminished the binding of anti-GP VI (FITC-JAQ1) mAb to human platelets, and it also reduced the coimmunoprecipitation of GP VI-bound Lyn after convulxin stimulation. The surface plasmon resonance results revealed that honokiol binds directly to GP VI, with a K_D_ of 289 μM. Platelet function analysis revealed that honokiol substantially prolonged the closure time in human whole blood and increased the occlusion time of thrombotic platelet plug formation in mice. In conclusion, honokiol acts as a potent antagonist of collagen GP VI in human platelets, and it has therapeutic potential in the prevention of the pathological thrombosis.

Platelets are anuclear blood cells from megakaryocyte that play a central role in hemostatic processes. Platelet activation and aggregation are also involved in thromboembolic disorders. When platelets are activated, they adhere to the injured blood vessel walls through the glycoprotein (GP) Ib–V–IX complex with von Willebrand factor (vWF) immobilized on the exposed subendothelial matrix^1^. Tethered platelets can then bind to collagen and initiate cellular activation processes. Activation signaling leads to a rise in the cytosolic Ca^+2^ concentration, cytoskeletal rearrangement, the release of granule contents, and the functional upregulation of integrin adhesion receptors enabling firm adhesion and thrombus growth. Thrombi that develop through this mechanism are the major source of many cardiovascular disorders (e.g., ischemic stroke, atherosclerosis, and myocardial infarction)^1^.

Platelet collagen receptors are grouped on the basis of their interaction with collagen. GP VI, integrin α_2_β_1_, and CD36 bind to collagen directly, whereas GP Ibα and integrin α_IIb_β_3_ interact with collagen-bound vWF^2^. Platelet GP VI is the major platelet collagen receptor in the formation of platelet aggregates on collagen surfaces under blood flow^3^. Integrin α_2_β_1_ is also a major collagen receptor on both endothelial cells and platelets^2^.

Honokiol (Fig. 1A) is the main biphenyl neolignan derived from Hou pu, the cortex of *Magnolia officinalis* (Magnoliaceae), which has been used for treating acute enteritis, bacterial and amebic diarrhea, chronic gastritis, and other diseases in the field of traditional Chinese medicine^4^. Recent studies have shown that honokiol possesses wide-ranging biological and clinically relevant properties, without an appreciable degree of toxicity^5^. Several studies have reported that honokiol induces cell-cycle arrest and apoptosis in cancer cells^6^,^7^ and inhibits tumor-associated lymphangiogenesis and metastasis in lung carcinoma models induced by vascular endothelial growth factor^8^. Zhang *et al*.^9^ discovered novel evidence for the anti-inflammatory effects of honokiol in the treatment of ischemic stroke by inhibiting NF-κB activation. Hoi *et al*.^10^ provided a scientific rationale for the clinical use of honokiol in the treatment of Alzheimer disease through attenuated amyloid β-induced cell death in rat adrenal medulla pheochromocytoma PC12 cells.

Moreover, Hu *et al*.^4^ reported that honokiol substantially inhibited collagen-stimulated rabbit platelet aggregation *ex vitro*, and prolonged the thrombus occlusion time in an electrical current-stimulated carotid thrombosis model in rats. Our preliminary findings similarly revealed that honokiol specifically inhibited collagen-stimulated washed human platelet aggregation. Despite the crucial role of platelets in the development of cardiovascular diseases, detailed data on the characteristics and functional activity of honokiol in platelet activation are relatively scant. The current study thus examined the cellular signaling events associated with honokiol-mediated platelet inhibition *ex vivo* and *in vivo.*

## Results

### Honokiol specifically inhibits washed human platelet aggregation stimulated by collagen, but not by other agonists

**Figure 1:**
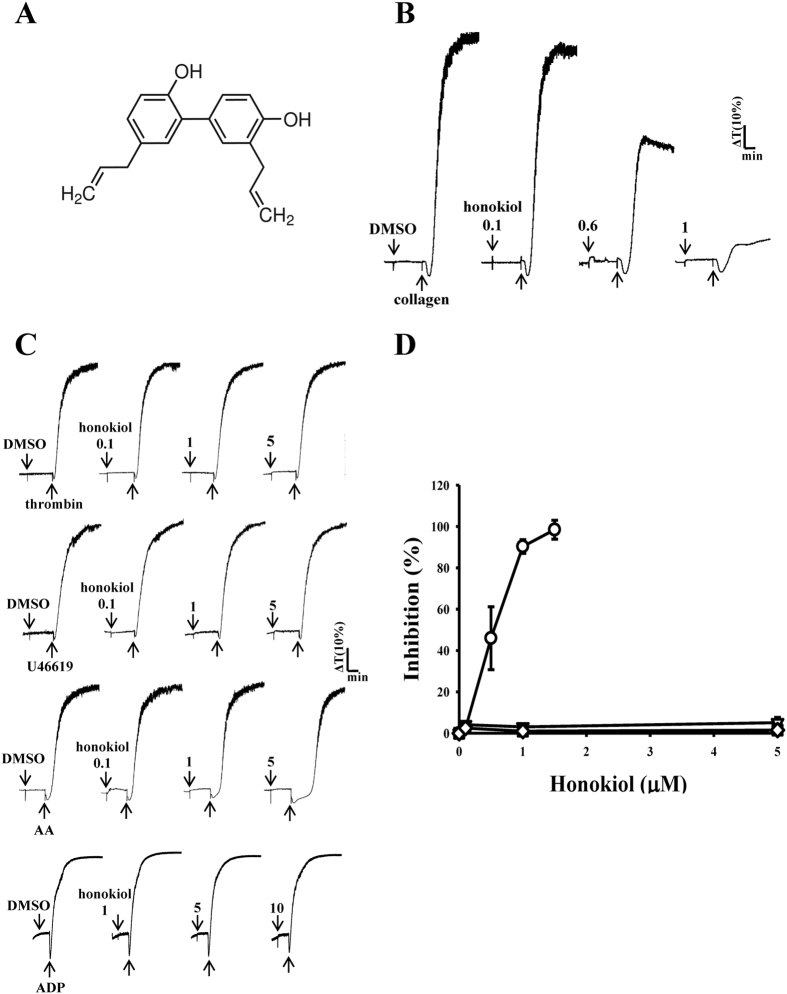
Concentration-dependent inhibitory effect of honokiol in agonist-stimulated platelet aggregation. Washed human platelets (3.6 × 10^8^ cells/ml) were preincubated with honokiol (0.1–5 μM) (**A**, chemical structure) or a solvent control (0.5% DMSO), and subsequently treated with 1 μg/ml of collagen (○), 0.05 IU/ml of thrombin (◇), 1 μM U46619 (□), or 60 μM arachidonic acid (∇) to trigger platelet aggregation for 6 min (**B–D**). For the other experiment, platelet-rich plasmas were preincubated with honokiol (1–10 μM), subsequently treated with 20 μM ADP to trigger platelet aggregation for 6 min (**C**). Statistical data (**D**) are presented as the means ± SEM (n = 4).

Platelet aggregation stimulated by 1 μg/ml of collagen was concentration-dependently inhibited by 0.1–1.0 μM honokiol (Fig. 1B). However, it did not affect platelet aggregation stimulated with other agonists, including 0.05 U/ml of thrombin, 1 μM U46619, or 60 μM AA, even when the concentration of honokiol used was up to 5 μM in this reaction (Fig. 1C,D). In addition, 1–10 μM honokiol had no effects in platelet aggregation stimulated by 20 μM ADP in platelet-rich plasmas (Fig. 1C). These results revealed that honokiol may specifically antagonize collagen-stimulated platelet activation. The 50% inhibitory concentration value of honokiol for collagen-stimulated platelet aggregation was approximately 0.6 μM (Fig. 1D). The solvent control (0.5% DMSO) did not significantly affect platelet aggregation (Fig. 1C). The aggregation curve of the platelets preincubated with 10 μM honokiol for 10 min and subsequently washed twice with Tyrode’s solution was found to have no significant differences against those of the platelets preincubated with 0.5% DMSO under equivalent conditions, indicating that the inhibitory effects of honokiol were reversible and noncytotoxic (data not shown). Moreover, the LDH study revealed that 10 μM honokiol incubated with the platelets for 20 min did not significantly increase LDH activity or exert cytotoxic effects on the platelets, even at concentrations of up to 50 μM (data not shown), indicating that honokiol did not affect platelet permeability or induce platelet cytolysis.

### Effects of honokiol on ATP release, the intracellular calcium level, and toxicity in zebrafish embryos

**Figure 2:**
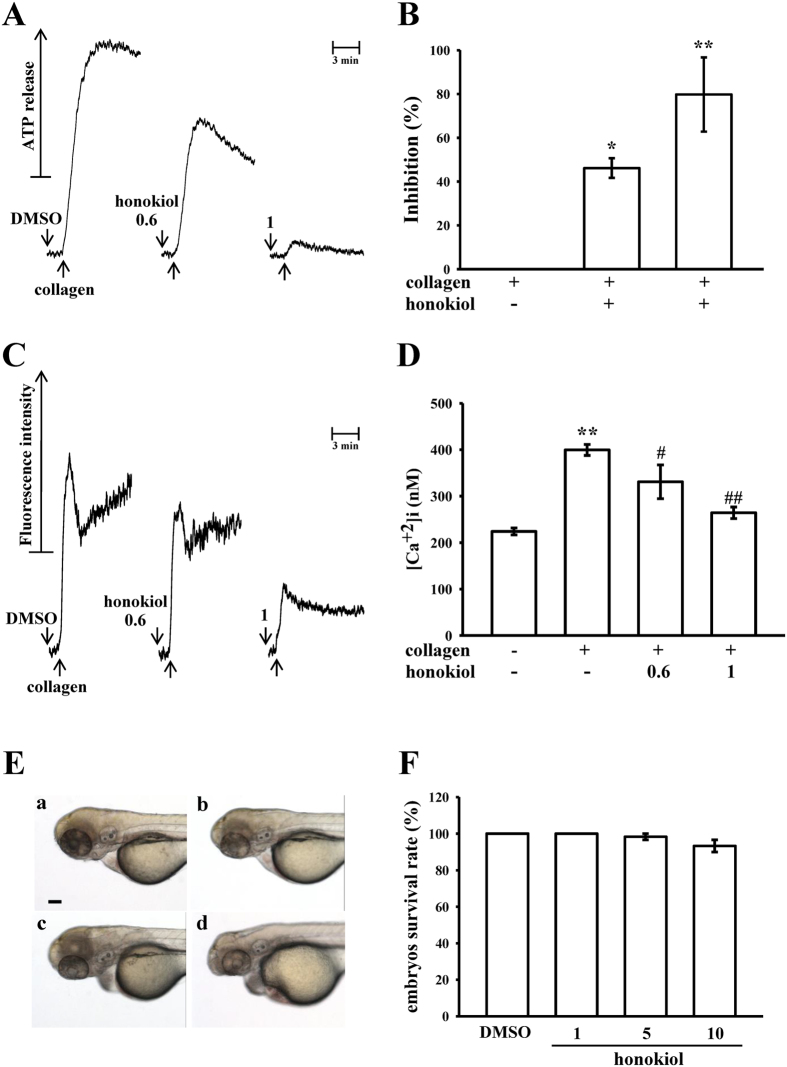
Honokiol in the ATP-release reaction, relative [Ca^2+^]i mobilization in human platelets, and toxicity in zebrafish embryonic development. Washed platelets (3.6 × 10^8^ cells/ml) were preincubated with honokiol (0.6 μM and 1 μM) or the solvent control (0.5% DMSO), after which 1 μg/ml of collagen was added to stimulate either the ATP-release reaction for 6 min (**A,B**) or to induce relative [Ca^2+^]i mobilization for 10 min (**C,D**). Data (**B,D**) are presented as the means ± SEM (n = 4). *p < 0.05 and **p < 0.01, compared with the control (resting) platelets; ^#^p < 0.05 and ^##^p < 0.01, compared with the collagen-treated platelets. For the other experiments (**E**), wild-type zebrafish embryos at 6 postfertilization were exposed to a solvent control (a, 0.5% DMSO) or various concentrations of honokiol (b, 1 μM; c, 5 μM; d, 10 μM) (scale bar 100 μm). Statistical data (**F**) show the means ± SEM (n = 60).

Because a previous study reported that granule secretions are critical markers of platelet activation prior to aggregation^11^, the present study examined whether honokiol affects collagen-stimulated platelet granule secretion. Consistent with the aggregation study, honokiol was found to concentration-dependently inhibit the collagen-induced ATP-release reaction in washed human platelets (Fig. 2A), and the corresponding statistical data are shown in Fig. 2B. Moreover, intracellular calcium ion [Ca^2+^]i play a critical role in platelet aggregation; that is, [Ca^2+^]i activates downstream signaling molecules and is thus the prerequisite for full platelet activation. Therefore, we analyzed the inhibitory effect of honokiol on Ca^2+^ mobilization in human platelets. Collagen (1 μg/ml) induced a relative increase in [Ca^2+^]i, which was concentration-dependently inhibited by 0.6 and 1.0 μM honokiol (resting, 224 ± 7 nM; collagen-activated, 399 ± 12 nM; 0.6 μM, 331 ± 37 nM; 1.0 μM, 264 ± 12 nM) (Fig. 2C,D).

To assess the honokiol toxicity potential at the organism level, zebrafish, a versatile *in vivo* vertebrate model used in many areas of biological investigation, were used in this study. We evaluated the toxicity of honokiol at ranges of 1–10 μM in wild-type zebrafish embryos treated for 6 dpf. The results obtained from this assay revealed nonsignificant phenotypic differences between the solvent control (0.5% DMSO) (Fig. 2Ea) and the honokiol (1, 5, and 10 μM)-treated zebrafish embryos throughout the experiment (n = 60) (Fig. 2Eb–d). No developmental defects or decreases in viability were observed in the zebrafish embryos in the presence of honokiol.

### Honokiol specifically inhibits platelet aggregation and the phosphorylation of Lyn, PLCγ2, and PKC stimulated with convulxin

**Figure 3:**
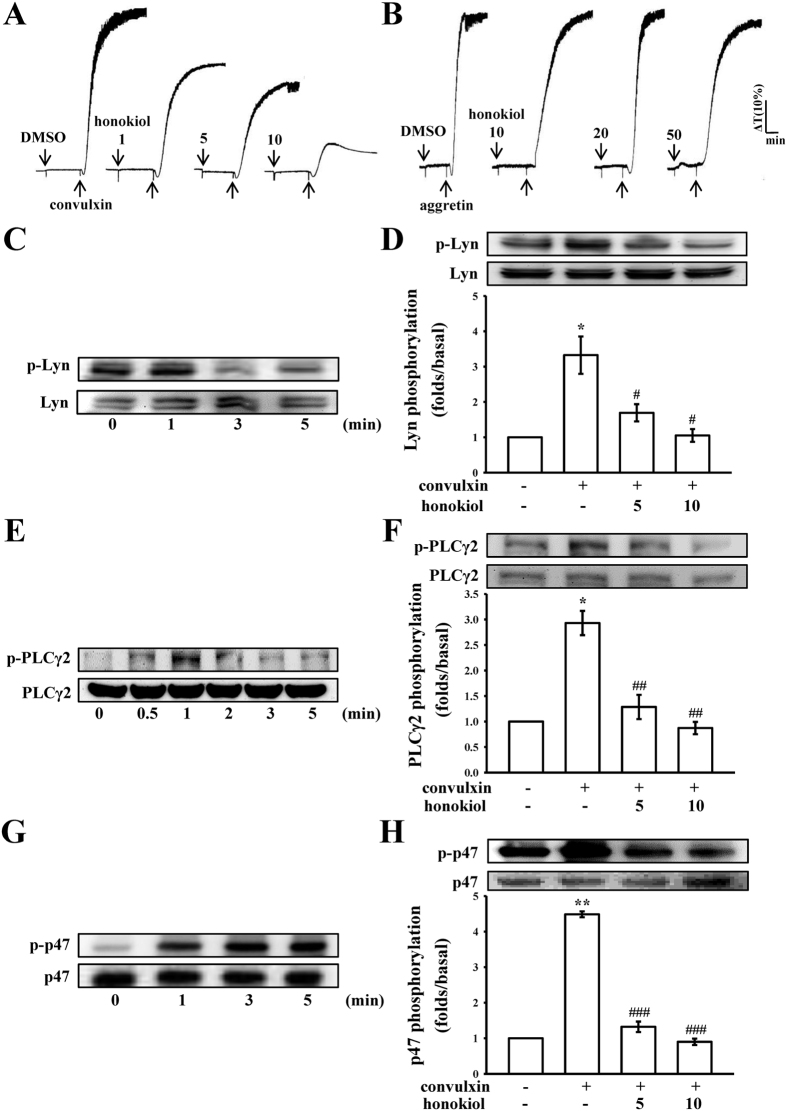
Effects of honokiol on platelet aggregation and Lyn, phospholipase Cγ2, and protein kinase C substrate (p47) phosphorylation stimulated with convulxin in washed human platelets. Washed platelets (3.6 × 10^8^ cells/ml) were preincubated with honokiol (1–10 μM) or the solvent control (0.5% DMSO), followed by the addition of 5 ng/ml of convulxin (**A**) or 1 μg/ml of aggretin (**B**) to trigger platelet activation. The profiles in A and B represent 4 independent experiments. Furthermore, washed platelets (1.2 × 10^9^ cells/ml) were preincubated with or without honokiol (5 μM and 10 μM) or the solvent control (0.5% DMSO), followed by the addition of 5 ng/mL of convulxin to trigger platelet activation at the indicated times (0.5 to 5 min). The cells were collected, and the subcellular extracts were analyzed for the presence of phosphorylated (**C,D**) Lyn, (**E,F**) phospholipase Cγ2 (PLCγ2), and (**G,H**) p47. Based on the convulxin’s maximum stimulation time points, immunoblot analysis was performed with convulxin stimulation of 1 min for phosphorylated (**D**) Lyn and (**F**) PLCγ2, and 3 min for (**H**) p47. Data are presented as the means ± SEM (n = 4). *p < 0.05 and **p < 0.01, compared with the control (resting) platelets; ^#^p < 0.05, ^##^p < 0.01 and ^###^p < 0.001, compared with the convulxin-treated platelets.

Integrin α_2_β_1_ and GP VI are major collagen receptors that mediate platelet adhesion and aggregation^12^,^13^,^14^. Treatment with 1–10 μM honokiol substantially inhibited platelet aggregation stimulated with 5 ng/ml of convulxin, a GP VI agonist, which is purified from the venom of *Crotalusdurissus terrificus*^13^, but not with aggretin (1 μg/ml), an integrin α_2_β_1_ agonist (aggretin also activates C-type lectin-like receptor 2)^12^,^15^, or even with 50 μM honokiol (Fig. 3A,B). Honokiol (1 μM) also substantially inhibited the ATP-release reaction stimulated by convulxin, but not by aggretin (data not shown), indicating that honokiol inhibits platelet activation through the GP VI, but not through integrin α_2_β_1_.

Lyn activation triggers a cascade of signaling events mediated by the Lyn phosphorylation of tyrosine residues within the immunoreceptor tyrosine-based activation motifs (ITAMs) of GP VI^16^. PLCγ2 hydrolyzes phosphatidylinositol 4,5-bisphosphate (PIP_2_) to generate the secondary messengers inositol 1,4,5-trisphosphate (IP_3_) and diacylglycerol (DAG). The DAG activates PKC, inducing p47 protein phosphorylation (47-kDa, pleckstrin) and ATP release. Thus, PLCγ2 plays a critical role as an upstream regulator of PKC activation in activated platelets^17^. As shown in Fig. 3C,E, convulxin (5 ng/ml) time-dependently triggered both Lyn and PLCγ2 phosphorylation, the maximal activation of which peaked at 1 min after convulxin stimulation. Honokiol (5 μM and 10 μM) obviously inhibited both Lyn and PLCγ2 phosphorylation at 1 min after convulxin stimulation (Fig. 3D,F). In addition, a protein with an apparent molecular weight similar to that of p47 (47-kDa) was predominantly phosphorylated within 3–5 min after stimulation with 5 ng/ml of convulxin compared with the protein profile of non-activated platelets (Fig. 3G). Treatment with 5 μM and 10 μM honokiol reduced the amount of the phosphorylated 47-kDa protein at 3 min in the convulxin-activated platelets (Fig. 3H).

### Inhibitory effect of honokiol on convulxin-stimulated MAPKs and Akt activation

**Figure 4:**
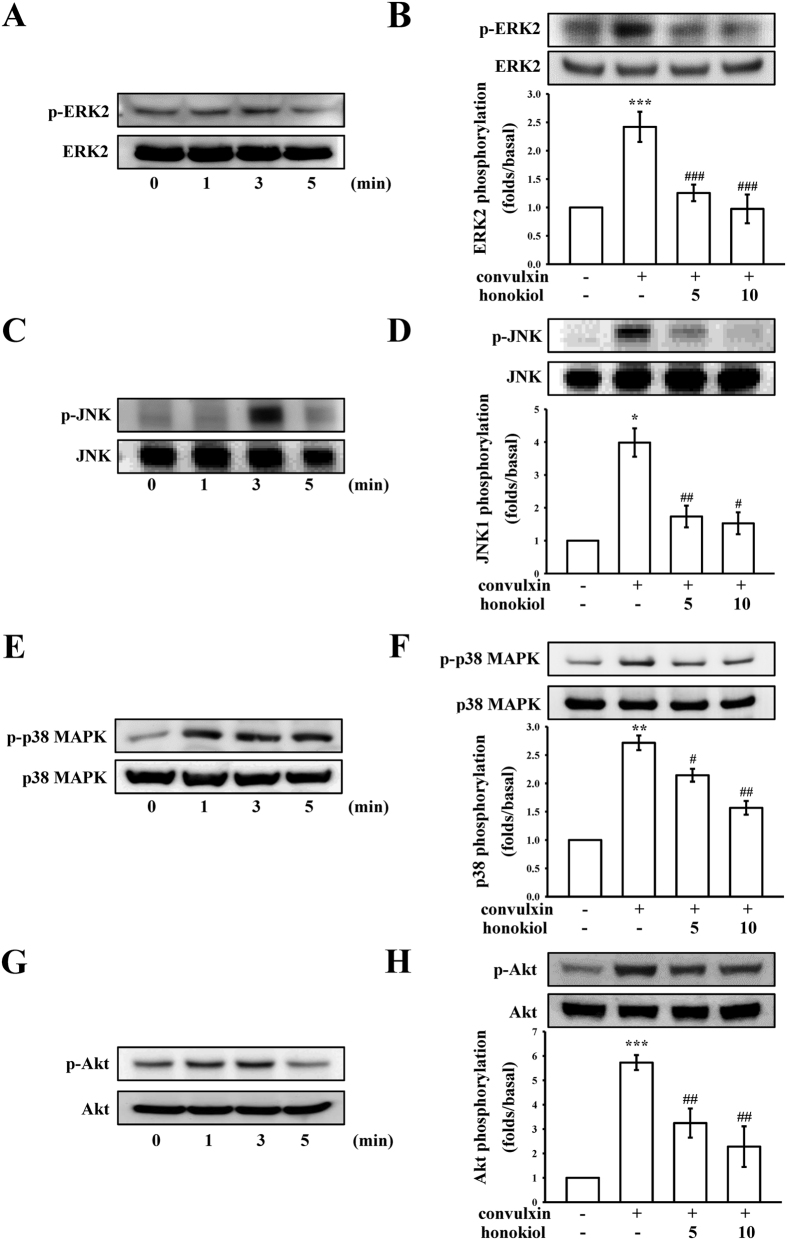
Effects of honokiol on ERK2, JNK, p38 MAPK, and Akt phosphorylation stimulated with convulxin. Washed platelets (1.2 × 10^9^ cells/ml) were preincubated with or without honokiol (5 μM and 10 μM) or the solvent control (0.5% DMSO), followed by the addition of 5 ng/ml of convulxin at the indicated times (1 to 5 min). Cells were collected, and the subcellular extracts were analyzed for the presence of phosphorylated (**A,B**) ERK2, (**C,D**) JNK, (**E,F**) p38 MAPK, and (**G,H**) Akt. Data are presented as the means ± SEM (n = 4). *p < 0.05, **p < 0.01 and ***p < 0.001, compared with the control (resting) platelets; ^#^p < 0.05, ^##^p < 0.01 and ^###^p < 0.001, compared with the convulxin-treated platelets.

To further investigate the inhibitory mechanisms of honokiol in convulxin-stimulated platelet activation, we detected several signaling molecules such as MAPKs (e.g., ERK1/2, JNK1/2, and p38 MAPK), which control major cellular responses in eukaryotic organisms as well as Akt, a Ser–Thr kinase with pleiotropic effects on cell survival, growth, and metabolism. As shown in Fig. 4, we found that 4 proteins (i.e., ERK2, JNK, p38 MAPK, and Akt) were maximally phosphorylated 3 min after convulxin stimulation, and were considerably inhibited with 5 μM and 10 μM honokiol.

### Honokiol binding to collagen GP VI on human platelets

**Figure 5:**
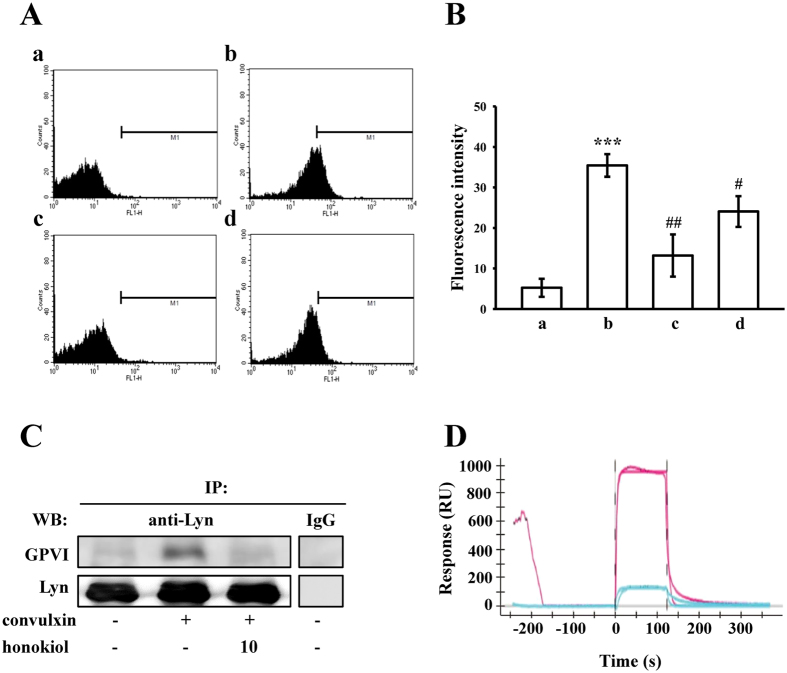
Association of honokiol with GP VI on human platelets. (**A**) The solid line represents the fluorescence profiles of the platelets in the presence of (a) FITC only (background) or (b) FITC-JAQ1 mAb (1 μg/ml); or preincubated with (c) convulxin (5 ng/ml) or (d) honokiol (10 μM), followed by the addition of FITC-JAQ1 mAb (1 μg/ml). Statistical graphs (**B**) show the means ± SEM (*n* = 4). ***p < 0.001, compared with the control (background) group; ^#^p < 0.05 and ^##^p < 0.01, compared with the FITC-JAQ1 mAb group. (**C**) For the immunoprecipitation study, washed platelets (1.2 × 10^9^ cells/ml) were preincubated with honokiol (10 μM) or the solvent control (0.5% DMSO) for 3 min, followed by the addition of convulxin (5 ng/ml) to trigger platelet activation. The proteins in the cellular extracts were then immunoprecipitated using the anti-Lyn pAb (1 μg/ml) or control IgG (1 μg/ml). The immunoprecipitates were then analyzed through immunoblotting using the anti-JAQ1 mAb and anti-Lyn pAb. The profile represents 4 independent experiments. (**D**) The direct binding of honokiol to GP VI was examined using surface plasmon resonance (SPR) technology, as described in the Methods section. In brief, the sensorgram showed the time course of the honokiol signal in response (RU) to treatment with 10 μM (blue line) and 100 μM honokiol (red line), and the binding data points were fitted using the Langmuir equation. The profile represents 4 independent experiments.

We further evaluated whether honokiol inhibits platelet activation by specifically binding to collagen GP VI on the platelet membrane. As shown in Fig. 5A,B, the relative fluorescence intensity of FITC-JAQ1 mAb (1 μg/ml) bound directly to the platelets was significantly higher than that of the resting counterparts (Fig. 5Aa,b), and FITC-JAQ1 mAb binding diminished markedly in the presence of convulxin (5 ng/ml) or honokiol (10 μM) (Fig. 5AC,d, respectively). The corresponding statistical data are shown in Fig. 5B. Moreover, honokiol (10 μM) had no effects on binding with FITC-anti-protease-activated receptor (PAR)-4 Ab (1 μg/ml) to thrombin PAR-4 receptor in human platelets stimulated by collagen (1 μg/ml) (data not shown). In addition, the proteins in the cellular extracts of the platelets were immunoprecipitated with anti-Lyn pAb (1 μg/ml), and the immunoprecipitates were analyzed through immunoblotting with the anti-GP VI mAb and anti-Lyn pAb. As shown in Fig. 5C, convulxin (5 ng/ml) obviously stimulated the association between Lyn and GP VI compared with the resting platelets, whereas treatment with 10 μM honokiol attenuated this association. To further identify the association of honokiol with GP VI, the SPR technique was employed. The GP VI from the total platelet lysates was captured using the anti-GP VI mAb immobilized on a GLH sensor chip. The binding response of 10 μM and 100 μM honokiol to GP VI is depicted in Fig. 5D (blue and red lines, respectively), and the fitted parameters were calculated using the Langmuir model (Ka: 7.41 × 10^2^ M^−1^s^−1^; Kd: 2.14 10^−1^ s^−1^; K_D_: 289 μM)^18^. Overall, these results revealed that honokiol binds directly to the collagen receptor GP VI on human platelets.

### Antithrombotic activities of honokiol *ex vivo* and *in vivo*

**Figure 6:**
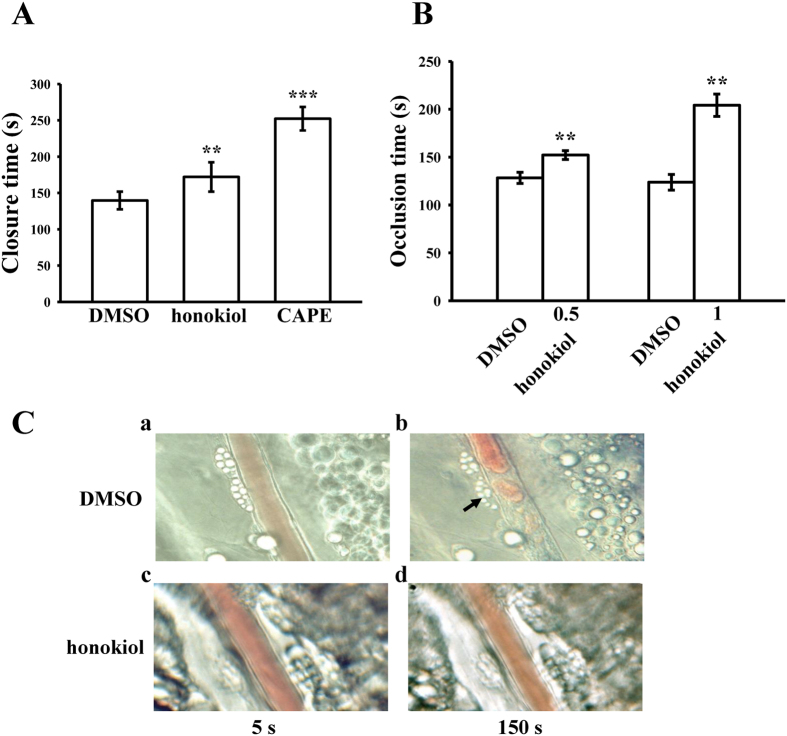
Honokiol on closure time determined through PFA-100 analysis and thrombotic platelet plug formation in the mesenteric venules of mice. (**A**) Shear-induced platelet plug formation in whole blood was determined according to closure time (CT), as described in the Methods section. The CT of CEPI for whole blood was recorded for the solvent control (0.5% DMSO), honokiol (10 μM), or CAPE (50 μM). Data are presented as the means ± SEM (n = 6). **p < 0.01 and ***p < 0.001, compared with the solvent control group. (**B**) Mice were administered an intravenous bolus of the solvent control (0.5% DMSO) or honokiol (0.5 or 1.0 mg/kg), and the mesenteric venules were irradiated to induce microthrombus formation (occlusion time). Data are presented as the means ± SEM (n = 6). **p < 0.01, compared with the 0.5% DMSO group. (**C**) Microscopic images (x400 magnification) of DMSO-treated controls (a,b) and honokiol (1.0 mg/kg)-treated groups (c,d) were recorded at 5 s (a,c) and 150 s (b,d) after irradiation. The photographs are representative examples of 6 similar experiments. The arrow indicates platelet plug formation.

In this study, we tested *ex vivo* shear-induced platelet plug formation in human whole blood. The PFA-100 instrument was used to mimic the *in vivo* conditions of blood vessel injury in humans, in whom platelets are exposed to a high shear rate. The CTs of CEPI in the solvent control (0.5% DMSO) were 140 ± 12 s (Fig. 6A). Treatment with 10 μM honokiol or 50 μM CAPE, which has been evidenced as a specific antagonist of collagen receptors^19^, significantly increased the CT of CEPI to 172 ± 20 s and 252 ± 16 s, respectively (n = 6) (Fig. 6A). Furthermore, we investigated the effect of honokiol on thrombus formation in mice. The occlusion time in microvessels pretreated with 15 μg/kg of fluorescein sodium was approximately 120 s. When honokiol was administered at 0.5 or 1.0 mg/kg after pretreatment with fluorescein sodium, the occlusion times were significantly prolonged compared with those of the 0.5% DMSO-treated controls (DMSO, 128 ± 6 s vs. 0.5 mg/kg, 152 ± 5 s, n = 6, p < 0.01; DMSO, 124 ± 8 s vs. 1.0 mg/kg, 204 ± 12 s, n = 6, p < 0.01) (Fig. 6B). The thrombotic platelet plug was observed in the mesenteric microvessels at 150 s, but not at 5 s, following irradiation in the DMSO-treated group (Fig. 6Ca/b). After the administration of 1.0 mg/kg of honokiol, platelet plug formation was not observed at either 5 or 150 s after irradiation (Fig. 6Cc/d). The blood flow rate of the DMSO-treated venule was lower than that of the honokiol-treated venule, because the platelet plug appeared at 150 s (Fig. 6Cb).

## Discussion

Our results revealed for the first time that honokiol exhibited potent and selective antiplatelet activity by binding to collagen GP VI on human platelets, thereby effectively inhibiting the convulxin-stimulated activation of platelets associated with Lyn, PLCγ2-PKC, MAPKs, and AKT activation *ex vivo*, and inhibited arterial thrombogenesis *in vivo*. The platelets are activated through various physiological stimuli (e.g., thrombin, ADP, and collagen). These agonists are believed to exert their effects by interacting with specific receptors on the platelet membrane. Collagen is present in the vascular subendothelium and vessel wall, and acts as both a substrate for platelet adhesion and an endogenous platelet activator. Integrin α_2_β_1_ and GP VI^13^ are platelet receptors known to interact directly with collagen. Signals (e.g., tyrosine phosphorylation and matrix remodeling) are activated in cells expressing integrin α_2_β_1_ after cell adhesion to collagen^20^.

**Figure 7:**
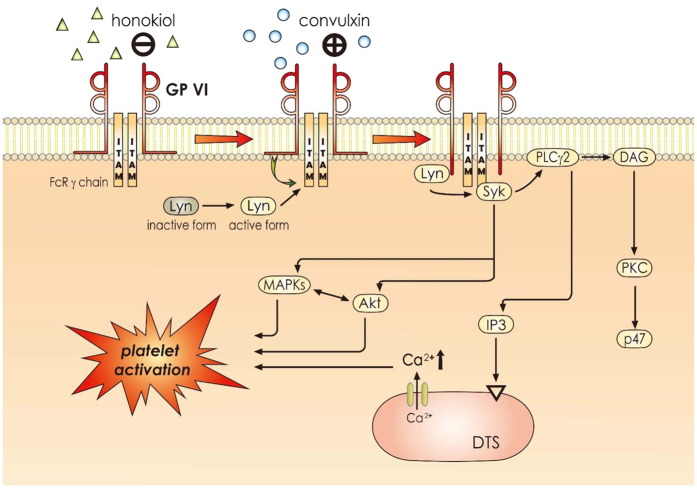
Hypothetical scheme of the inhibitory signaling of honokiol in platelet activation. Honokiol binds to GP VI before interfering with the activation of convulxin-mediated signal events, such as Lyn phosphorylation and phospholipase Cγ2 (PLCγ2)-diacylglycerol (DAG)-protein kinase C (PKC)-p47, MAPKs, and Akt signaling pathways. It subsequently inhibits [Ca^+2^]i mobilization and ultimately inhibiting platelet activation. DTS, dense tubular system; IP_3_, inositol 1,4,5-trisphosphate; ITAM, immunoreceptor tyrosine-based activation motif.

GP VI is a 60–65 kDa type I transmembrane glycoprotein of the immunoglobulin superfamily^21^. GP VI ligands, including collagen and convulxin, induce receptor clustering, which facilitates the phosphorylation of the tandem tyrosines found in the ITAMs of the noncovalently associated Fcγ-chain receptor (FcRγ) adaptor by Src-family tyrosine kinases (e.g., Fyn and Lyn) (Fig. 7)^22^,^23^. Src was the first proto-oncogenic nonreceptor tyrosine kinase characterized in humans. Both Fyn and Lyn are involved in GP VI-dependent platelet activation, and may phosphorylate FcRγ^24^. The cross-linking of the GP VI–FcRγ complex in platelets places GP VI-bound Fyn or Lyn in proximity to FcRγ, facilitating the phosphorylation of the FcRγ ITAM. This triggers the phosphorylation of downstream signals, leading to the activation of a kinase cascade (e.g., the PLCγ2-PKC-mediated pathways) (Fig. 7).

The activation of PLC results in IP_3_ and DAG production, which activates PKC, inducing the phosphorylation of the p47 protein (pleckstrin)^25^. The PKC activation enables specific responses that facilitate the transmission of particular activating signals in distinct cellular compartments. PLC enzymes can be classified into 6 families, where the PLCγ family comprises isozymes 1 and 2. PLCγ2 is involved in collagen-dependent signaling in platelets^26^. In our study, honokiol attenuated convulxin-induced PLCγ2-PKC activation; however, it did not reduce PDBu-induced PKC activation, indicating that honokiol had no direct effects on PKC activation.

Three MAPK subfamilies have been characterized extensively in mammalian cells: ERK1/2 (also called p44/42 MAPK), JNK1/2, and p38 MAPK. Specific MAPKKs (or MEKs) activate all of these kinases^27^. ERK1/2 is involved in cell proliferation, adhesion, and progression^28^. By contrast, p38 MAPK and JNK1/2 appear to be involved in apoptosis, whereas ERK2, JNK1, and p38 MAPK have all been identified in platelets^28^. The pathophysiological roles of JNK1 and ERK2 in platelets are unclear, but evidence implies that the suppression of integrin α_IIb_β_3_ activation may be involved^29^. In addition, p38 MAPK provides a crucial signal in collagen-induced platelet aggregation. We observed that SB203580, a p38 MAPK inhibitor, inhibited collagen-induced platelet aggregation substantially (data not shown). Among the numerous downstream targets of p38 MAPK, cytosolic phospholipase A_2_, which catalyzes AA release to produce thromboxane A_2_, is the most physiologically relevant^30^. Thus, MAPKs, especially p38 MAPK, appear to play a crucial role in platelet activation. In addition, Akt (also called protein kinase B) is a downstream effector of PI3-kinase^31^, and Akt-knockout mice have exhibited defects in agonist-induced platelet activation^32^. Three mammalian Akt isoforms exist: Akt 1, 2, and 3^33^. The first 2 were detected in human platelets^33^. Studies using Akt inhibitors in human platelets typically support a similar role for Akt 1 and 2 in human platelet activation. Therefore, protein kinases contributing to Akt activation, especially PI3-kinase β, may be desirable targets for the development of antithrombotic therapeutics. In our previous study, we found that both PI3-kinase/Akt and MAPKs (e.g., p38 MAPK) are mutually activated as the upstream regulators of PKC in activated platelets (Fig. 7)^34^. In the time-course study, we found the maximal phosphorylation of both Lyn and PLCγ2 within 1 min after convulxin stimulation, whereas that of the other signaling molecules including PKC, MAPKs, and Akt was found within 3 min. These results are consistent with those regarding the signaling cascades of GP VI activation (Fig. 7).

In this study, honokiol interfered with the binding of FITC-JAQ1 mAb to platelet membranes, and also abolished the IP of Lyn with GP VI after convulxin stimulation. SPR analysis revealed that honokiol directly binds to GP VI with a K_D_ value of approximately 289 μM; K_D_ is the equilibrium dissociation constant, which is a ratio of Kd/Ka between the receptor and the ligand. Studies have reported that the K_D_ values of collagen and convulxin binding to GP VI are approximately 41.7 nM and 0.4–4 nM, respectively^13^,^35^. A comparison of the K_D_ values showed that convulxin had a seemingly 10- to 100-fold higher binding affinity compared with collagen toward GP VI. This result also explains why honokiol required a higher concentration for inhibiting platelet aggregation stimulated with convulxin than for inhibiting that stimulated with collagen (10 μM vs. 1 μM). In addition, we also observed that honokiol (10 μM) obviously diminished Lyn phosphorylation at 1 min after convulxin (5 ng/ml) stimulation under unstirred condition in the presence of apyrase (2 U/ml), indomethacin (10 μM), and triflavin (1 μg/ml; inhibitor of integrin α_IIb_β_3_), as compared with the protein profile of non-activated platelets (please see Supplementary Fig. S1). This result indicated that honokiol antagonizes the GP VI-mediated signaling may not result from interfering the amplification of platelet activation (such as ADP release, thromboxane A_2_ formation, and outside-in signaling of the integrin α_IIb_β_3_). Moreover, the observed data of honokiol (1 μM) significantly diminished the binding of FITC-triflavin (1 μg/ml) to the integrin α_IIb_β_3_ in collagen-activated platelets, indicating that honokiol antagonizes the GP VI-mediated inside-out signaling in subsequent to interfere the activation of integrin α_IIb_β_3_ (please see Supplementary Fig. S2).

After endothelial cell injury, the exposure of subendothelial collagen is the major trigger that initiates platelet adhesion and aggregation at the site of injury. To mimic the clinical conditions of arterial thrombosis, we employed the PFA-100 instrument to determine the time required for platelet aggregation to occlude an aperture in collagen-coated membranes. Goto *et al*.^36^ reported that platelet adhesion to collagen is dependent on GP VI under flow conditions, and mouse platelets depleted of GP VI could not adhere to collagen under flow conditions^37^. In the current study, honokiol significantly prolonged CT *ex vivo* and thrombus formation in mice, which may have resulted from honokiol specifically interfering in the interaction between collagen and GP VI. Besides inhibition of platelet GP VI activation, other effects of honokiol could also lead to a prolongation of the occlusion time as Hu *et al*.^4^ reported that honokiol prolonged the thrombus occlusion time in rats through protection of endothelial cells and stimulation of prostacyclin and nitric oxide release. Our findings are consistent with those reported in a previous study, in which mice depleted of GP VI were completely protected from collagen-induced pulmonary thromboemboli^38^. In addition, the tail transection model of mice was used to examine the influence of honokiol on bleeding time *in vivo*. Aspirin is the most effective antiplatelet drug prescribed for the prevention or treatment of cardiovascular and cerebrovascular diseases, whereas it has unwanted prolongation of the bleeding time. In the tail transection model of mice, aspirin was administered (i.p.) as positive control at 1 mg/kg after 30 min markedly prolonged the bleeding time from 226.5 ± 21.3 s (PBS-treated control group; *n* = 8) to 413.8 ± 26.3 s (*n* = 8, p < 0.001; data not shown). The bleeding time of the honokiol (1 mg/kg)-treated mice was not prolonged as compared with the solvent control (0.5% DMSO)-treated mice (219.8 ± 18.7 s vs 258.6 ± 24.5 s, *n* = 8, p > 0.05; data not shown), indicating honokiol at 1 mg/kg obviously prolonged the occlusion time but without causing bleeding tendency in mice. Thus, platelet collagen receptors, especially GP VI, have emerged as targets of interest for novel antiplatelet drugs.

An essential role of platelets in cardiovascular diseases and cardiac mortality has been demonstrated in several landmark clinical trials, in which the inhibition of platelet activation and/or aggregation improved cardiovascular outcomes^39^. To the best of our knowledge, this study presented a novel finding; that is, honokiol, a plant-based natural product, acts as a potent antagonist of collagen GP VI. Honokiol may have therapeutic potential in the prevention of pathologic thrombi associated with coronary and cerebral artery diseases. However, our experiments did not dismiss the possibility that other as-yet-unidentified mechanisms (i.e., inhibition of GP VI clustering) might be involved in the honokiol-mediated inhibition of platelet activation^40^.

## Materials and Methods

### Chemicals and reagents

Honokiol (≧98%), collagen (type I from bovine skin), luciferin–luciferase, heparin, arachidonic acid (AA), U46619, thrombin, caffeic acid phenethyl ester (CAPE), prostaglandin E_1_ (PGE_1_), and bovine serum albumin (BSA) were purchased from Sigma-Aldrich (St. Louis, MO, USA). Fura 2-AM was obtained from Molecular Probe (Eugene, OR, USA). The anti-phospholipase Cγ2 (PLCγ2), anti-phospho (Tyr^759^) PLCγ2, anti-phospho-(Ser) protein kinase C (PKC) substrate, anti-phospho-p38 mitogen-activated protein kinase (MAPK) (Ser^180^/Tyr^182^), and anti-phospho-p44/p42 extracellular signal-regulated kinase (ERK) (Thr^202^/Tyr^204^) polyclonal antibodies (pAbs), as well as the anti-p38 MAPK, anti-phospho-c-Jun N terminal kinase (JNK) (Thr^183^/Tyr^185^), anti-phospho-Akt (Ser^473^), and anti-Akt monoclonal antibodies (mAbs) were purchased from Cell Signaling (Beverly, MA, USA). The protein A/G plus-agarose, convulxin, anti-Lyn, and anti-phospho Lyn pAbs were purchased from Santa Cruz (Santa Cruz, CA, USA). The anti-α-tubulin mAb was obtained from NeoMarkers (Fremont, CA, USA). The anti-GP VI (JAQ1) and fluorescein isothiocyanate (FITC)-anti-GP VI (FITC-JAQ1) mAbs were obtained from Emfret Analytics (Würzburg, Germany). The Hybond-P polyvinylidene difluoride (PVDF) membrane, an enhanced chemiluminescence (ECL) western blotting detection reagent and analysis system, horseradish peroxidase (HRP)-conjugated donkey anti-rabbit IgG, and sheep anti-mouse IgG were obtained from Amersham (Buckinghamshire, UK). Rabbit IgG control was obtained from GenScript (Piscattaway, NJ, USA). The Dade Behring PFA collagen/epinephrine (CEPI) test cartridge was obtained from Siemens Healthcare (Erlangen, Germany). Professor Tur Fu Huang at the Department of Pharmacology (College of Medicine, National Taiwan University) kindly provided aggretin for our experiments. Honokiol was dissolved in 0.5% dimethyl sulfoxide (DMSO) and stored at 4 °C until use.

### Platelet aggregation

Our study was approved by the Institutional Review Board of Taipei Medical University (TMU-JIRB-No.201101005) and conformed to the directives of the Helsinki Declaration. All human volunteers provided informed consent. Human platelet suspensions were prepared as described in a past study^41^. Blood was collected from healthy human volunteers (age: 20–30 y; none of the participants had a history of abnormal bleeding, diabetes mellitus, or arterial or venous thrombotic disorders, as determined with an extensive questionnaire) who had taken no medication during the 2 weeks prior to collection; the blood was mixed with an acid–citrate–dextrose solution. After centrifugation, the supernatant (platelet-rich plasma) was supplemented with 0.5 μM prostaglandin E_1_ and 6.4 IU/ml of heparin. Washed platelets were suspended in Tyrode’s solution containing 3.5 mg/ml of BSA. The final concentration of Ca^2+^ in the solution was 1 mM.

A lumi-aggregometer (Payton Associates, Scarborough, ON, Canada) was used to measure platelet aggregation, as described in a past study^41^. The platelet suspensions (3.6 × 10^8^ cells/ml) were preincubated with various concentrations of honokiol or an isovolumetric solvent control (final concentration, 0.5% DMSO) for 3 min before the addition of agonists (i.e., collagen, thrombin). The reaction was left to proceed for 6 min, and the extent of aggregation was expressed in light transmission units. To measure ATP release, 20 μl of a luciferin–luciferase mixture was added 1 min before the addition of collagen, and the amount of ATP released was compared against that released by the control.

### Determination of lactate dehydrogenase

Washed platelets (3.6 × 10^8^ cells/ml) were preincubated with 5 μM or 10 μM honokiol or a solvent control (0.5% DMSO) for 20 min at 37 °C. An aliquot of the supernatant (10 μl) was deposited on a Fuji Dri-Chem slide LDH-PIII (Fuji, Tokyo, Japan), and the absorbance wavelength was read at 540 nm with an ultraviolet-visible spectrophotometer (UV-160; Shimazu, Japan). A maximal value of lactate dehydrogenase (LDH) was observed in the sonicated platelets.

### Measurement of intracellular [Ca^+2^] ion mobilization with Fura 2-AM fluorescence

Citrated whole blood was centrifuged at 120 × *g* for 10 min. The supernatant was incubated with 5 μM Fura 2-AM for 1 h. Human platelets were prepared as described in the section “**Platelet aggregation**”. The platelet suspensions were adjusted to 1 mM Ca^2+^. The relative intracellular Ca^+2^ ion ([Ca^2+^]i) concentration was measured with a Jasco CAF 110 fluorescence spectrophotometer (Tokyo, Japan), operating at excitation wavelengths of 340 nm and 380 nm as well as an emission wavelength of 500 nm^41^.

### Zebrafish toxicity test

Zebrafish (*Danio rerio*) were obtained from the zebrafish core facility of Taipei Medical University and maintained on a 14-h light/10-h dark cycle. The Taipei Medical University Institutional Animal Care and Utilization Committee approved the study and the guidelines described in *The Zebrafish Boo*^42^ were follwed to the animal procedures. The zebrafish embryos were treated with various concentrations of honokiol at 20 h at 28 °C postfertilization for 144 h to evaluate the toxic effects of honokiol on the zebrafish embryos. Sixty dechorionated embryos were treated with 2 ml of honokiol (1, 5, or 10 μM) or a solvent control (0.5% DMSO) in a 24-well chamber. The treated embryos were observed at 2, 3, 4, 5, or 6 days postfertilization (dpf). At 6 dpf, the percentage of embryos exhibiting developmental abnormalities as well as the survival rate were determined. During exposure, photographs of the embryos were examined with an Olympus IX70-FLA inverted fluorescence microscope (Olympus, Tokyo, Japan). The images were taken using a SPOT digital camera system (Diagnostic Instruments, Sterling Heights, MI, USA) and assembled using Image J software^43^.

### Immunoblotting

Washed platelets (1.2 × 10^9^ cells/ml) were preincubated with 5 or 10 μM honokiol or 0.5% DMSO for 3 min, and convulxin (5 ng/ml) was subsequently added to trigger platelet activation. The reaction was stopped, and the platelets were immediately resuspended in 200 μl of a lysis buffer. Samples containing 80 μg of protein were separated on a 12% acrylamide gel through sodium dodecylsulfate polyacrylamide gel electrophoresis, and the protein was electrotransferred to PVDF membranes by using a Bio-Rad semidry transfer unit (Hercules, CA, USA). The blots were blocked with TBST (10 mM Tris-base, 100 mM NaCl, and 0.01% Tween 20) containing 5% BSA for 1 h and probed with various primary antibodies. The membranes were incubated with HRP-linked anti-mouse IgG or anti-rabbit IgG (diluted at 1:3000 in TBST) for 1 h. An ECL system was used to detect the immunoreactive bands, and the optical density was quantified using Bio-profil Biolight software (version 2000.01, Vilber Lourmat, Marne-la-Vallée, France).

### Flow cytometry

The FITC labeling of the anti-GP VI mAb (FITC-JAQ1 mAb) was performed in the current study. In brief, the platelets (3.6 × 10^8^ cells/ml) were preincubated with 5 ng/ml of convulxin or 10 μM honokiol for 3 min, followed by the addition of 1 μg/ml of FITC-JAQ1 mAb. The suspensions were analyzed in a Beckman Coulter flow cytometer (Brea, CA, USA). Data were collected from 50 000 platelets per experimental group, and the platelets were identified with their characteristic forward and orthogonal light-scattering profiles. All of the experiments were performed at least 4 times to ensure reliability.

### Immunoprecipitation

In brief, washed platelets (1.2 × 10^9^/ml) were preincubated with 10 μM honokiol or a solvent control (0.5% DMSO) for 3 min, followed by the addition of 5 ng/ml of convulxin for 1 min. They were then lysed in an immunoprecipitation (IP) buffer as described in a past study^44^. An equal amount of protein from each supernatant was precleared with protein A/G-agarose-conjugated beads for 2 h. The samples were then rotated overnight with the anti-Lyn pAb (1 μg/ml) or control IgG (1 μg/ml). The following day, 20 μl of beads was added and rotated overnight. Immunoprecipitates were washed 3 times before they were analyzed through immunoblotting with 20% acrylamide gel.

### Surface plasmon resonance analysis

Surface plasmon resonance (SPR) analysis was performed as described by Balducci *et al*.^18^, with minor modifications, using the ProteOn XPR36 Protein Interaction Array system (Bio-Rad). The system contains 6 parallel flow channels that uniformly immobilize up to 6 strips of ligands on the sensor surface. The running buffer was phosphate-buffered saline (PBS) with 0.005% Tween 20 with a pH of 7.4, and the experiments were performed at 25 °C. The anti-GP VI mAb (5 μg/ml) was immobilized on the GLH sensor chip (Bio-Rad) through amine coupling, and GP VI was captured from the total platelet lysates (3.6 × 10^8^ cells/ml). For the binding measurements, 10 or 100 μM honokiol or PBS (i.e., the reference buffer) was injected, and the association rate constant (Ka), dissociation rate constant (Kd), and equilibrium dissociation (binding) constant (K_D_) were determined from the sensorgram data (time course of the SPR signal) by fitting the data set with the simplest 1:1 interaction model by using ProteOn analysis software.

### Platelet function analysis in whole blood

A Dade Behring platelet function analysis system (PFA-100, Marburg, Germany) was used to measure platelet function^45^. Whole blood was preincubated with 0.5% DMSO, 50 μM CAPE, or 10 μM honokiol for 2 min, aliquots of whole blood (0.8 ml/cartridge) were then applied to the cartridges containing a CEPI-coated membrane before the contents were exposed to high-shear-flow conditions (5000–6000/s). The closure time (CT) was defined as the time required for the platelet plug to occlude the aperture in the membrane^45^.

### Measurement of sodium fluorescein-induced platelet thrombus formation in mouse mesenteric microvessels

Male ICR mice (age: 6 wk) were anesthetized using a mixture containing 75% air and 3% isoflurane maintained in 25% oxygen, and the external jugular vein was cannulated with a PE-10 tube for the intravenous administration of the dye and drugs^46^. Venules (30–40 μm) were irradiated at wavelengths <520 nm to produce a microthrombus. Two doses of honokiol (0.5 and 1 mg/kg) were administered 1 min after sodium fluorescein (15 μg/kg) administration, and the time required for the thrombus to occlude the microvessel (occlusion time) was recorded. In this experiment, the thrombogenic animal model was used in accordance with the Guide for the Care and Use of Laboratory Animals (8th edition, 2011) and was approved by Affidavit of Approval of Animal Use Protocol-Taipei Medical University (LAC-2014-0043).

### Data analysis

The experimental results are expressed as the means ± SEM. The values of n refer to the number of experiments, each conducted with different blood donors. Paired Student *t* tests were performed to determine the significant differences between the data for each group in the *in vivo* experiments. Data obtained from the other experiments were analyzed with analysis of variance (ANOVA). If the ANOVA results revealed a significant difference between the group means, the Newman–Keuls method was used for further comparison. Comparison results with a p value lower than 0.05 were considered statistically significant. All statistical analyses were performed using the SAS (version 9.2) software package (SAS Inc., Cary, NC, USA).

## Additional Information

**How to cite this article**: Lee, T.-Y. *et al*. Honokiol as a specific collagen receptor glycoprotein VI antagonist on human platelets: Functional ex vivo and in vivo studies. *Sci. Rep.* **7**, 40002; doi: 10.1038/srep40002 (2017).

**Publisher's note:** Springer Nature remains neutral with regard to jurisdictional claims in published maps and institutional affiliations.

## Supplementary Material

Supplementary Information
