# Supplementary material for: Honokiol as a specific collagen receptor glycoprotein VI antagonist on human platelets: Functional ex vivo and in vivo studies
Source: Sci Rep. 2017 Jan 5;7:40002. doi: 10.1038/srep40002 (PMC5213647; doi:10.1038/srep40002)
Supplement: Supplementary Information [file srep40002-s1.pdf]

Supplementary Information

Honokiol as a specific collagen receptor glycoprotein VI antagonist on human platelets: Functional ex vivo and in vivo studies

Tzu-Yin Lee, Chao-Chien Chang, Wan-Jung Lu, Ting-Lin Yen, Kuan-Hung Lin, Pitchairaj Geraldine, Jiun-Yi Li, and Joen-Rong Sheu

Fig. S1. Effects of honokiol on Lyn phosphorylation in unstirred human platelets stimulated by convulxin.

Fig. S2. Effect of honokiol on the inhibition of FITC-triflavin binding to the integrin $\alpha_{IIb}\beta_3$ in activated platelets.

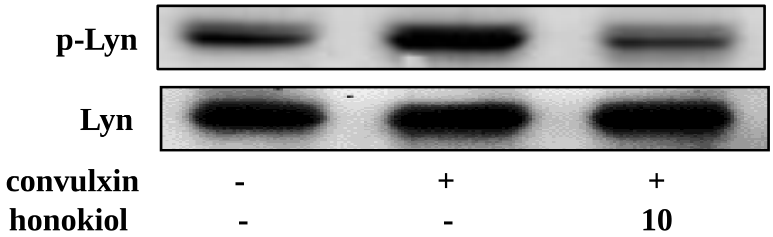

Fig. S1. Effects of honokiol on Lyn phosphorylation in unstirred human platelets stimulated by convulxin. Washed platelets (3.6 × 10^8 cells/ml) were preincubated with apyrase (2 U/ml), indomethacin (10 μM), and triflavin (1 μg/ml) with or without honokiol (10 μM) or the solvent control (0.5% DMSO) for 5 min, followed by the addition of 5 ng/ml of convulxin to trigger Lyn phosphorylation for 1 min. The profile represents 3 independent experiments.

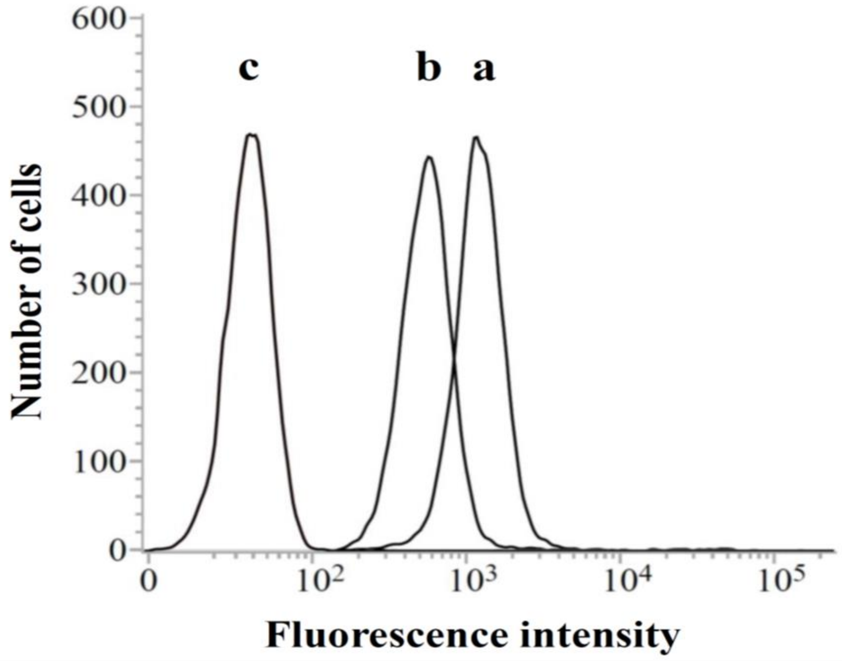

Fig. S2. Effect of honokiol on the inhibition of FITC-triflavin binding to the integrin $\alpha_{IIb}\beta_3$ in activated platelets. Washed platelets (3.6×10^8/ml) were preincubated with (a) 0.5% DMSO, (b) honokiol (1 μM), or (c) EDTA (2 mM; negative control), followed by the addition of FITC-triflavin (1 μg/ml) and subsequently treated with collagen (1 μg/ml) to trigger platelet activation. The profile represents 3 independent experiments.
